# Effects of Continuous Infusion Therapies on Non-Motor Symptoms in Advanced Parkinson’s Disease: A Systematic Review

**DOI:** 10.3390/brainsci16070698

**Published:** 2026-06-30

**Authors:** Domiziana Rinaldi, Lanfranco De Carolis, Claudia Ledda, Silvia Galli, Morena Giovannelli, Alberto Romagnolo, Maurizio Zibetti, Marco Salvetti, Leonardo Lopiano, Gabriele Imbalzano

**Affiliations:** 1 Department of Neuroscience, Mental Health and Sensory Organs (NESMOS), Sapienza University of Rome, 00189 Rome, Italy; 2 Department of Neuroscience “Rita Levi Montalcini”, University of Turin, 10126 Turin, Italy; 3 Struttura Complessa Neurologia 2U, Azienda Ospedaliera Universitaria Città della Salute e della Scienza, 10126 Turin, Italy

**Keywords:** Parkinson’s disease, non-motor symptoms, infusion therapy, levodopa–carbidopa intestinal gel, apomorphine, foslevodopa/foscarbidopa, subcutaneous, device-aided therapies, sleep disorders, autonomic symptoms

## Abstract

**Highlights:**

**What are the main findings?**

- Continuous infusion therapies in advanced Parkinson’s disease were associated with improvement in global non-motor burden, particularly for sleep/fatigue and gastrointestinal symptoms.
- Evidence was strongest for levodopa–carbidopa intestinal gel (LCIG), while data for CSAI, LECIG, and subcutaneous levodopa formulations remain limited.

**What are the implications of the main findings?**

- Non-motor symptoms should be systematically considered when selecting and monitoring infusion therapies in advanced Parkinson’s disease.
- Future studies should include standardized domain-specific non-motor assessments and longer follow-up, particularly for newer infusion therapies.

**Abstract:**

**Background/Objectives**: Non-motor symptoms (NMS) are highly prevalent in advanced Parkinson’s disease (PD) and substantially affect quality of life. Continuous infusion therapies are established treatment options for motor fluctuations not controlled by oral medication, but their effects on NMS remain incompletely characterized. We aimed to evaluate the effects of continuous infusion therapies on NMS in advanced PD. **Methods**: A systematic review was conducted according to PRISMA guidelines. PubMed, Embase, and Cochrane were searched for English-language original studies published between January 2005 and 1 March 2026. Eligible studies included patients with PD treated with levodopa–carbidopa intestinal gel (LCIG), continuous subcutaneous apomorphine infusion (CSAI), subcutaneous levodopa formulations, or levodopa–entacapone–carbidopa intestinal gel (LECIG) and reported quantitative NMS outcomes. Due to methodological heterogeneity, results were synthesized qualitatively. **Results**: Fifty-four studies were included. Most evaluated LCIG (*n* = 38), followed by CSAI (*n* = 14), subcutaneous levodopa formulations (*n* = 6), and LECIG (*n* = 2). Overall, 4157 patients were assessed at baseline and 2919 at follow-up. Global non-motor burden improved in 33/45 (73.3%) baseline-to-follow-up comparisons. NMSS total score decreased from 84.4 ± 35.2 to 54.9 ± 17.6. The most consistent benefits were observed for sleep/fatigue and gastrointestinal symptoms. Sleep/fatigue outcomes improved in 26/31 (83.9%) baseline-to-follow-up comparisons. Cognitive outcomes were mostly stable, while cardiovascular, urinary, sexual, and mood-specific outcomes showed less consistent benefit. **Conclusions**: Continuous infusion therapies may be associated with reduced global non-motor burden in advanced PD, particularly sleep/fatigue and gastrointestinal symptoms. Evidence is strongest for LCIG, while data for CSAI, LECIG, and subcutaneous levodopa formulations remain limited.

## 1. Introduction

Parkinson’s disease (PD) is a progressive neurodegenerative disorder characterized by motor symptoms and a wide range of non-motor symptoms (NMS). These include sleep disorders, cognitive impairment, mood and affective disturbances, autonomic dysfunction (including constipation, orthostatic hypotension, urinary and sexual dysfunction), and sensory symptoms, such as hyposmia and pain [1]. NMS are highly prevalent across all disease stages and often represent a major determinant of reduced quality of life, caregiver burden, and disability [2,3].

As PD progresses, NMS may become increasingly prominent and may fluctuate throughout the day, often in parallel with motor fluctuations [4]. The progressive degeneration of nigrostriatal dopaminergic neurons leads to a gradual loss of the physiological buffering capacity for exogenous levodopa, increasing patients’ dependence on fluctuating plasma drug concentrations. Consequently, the short half-life of oral levodopa, together with variable gastrointestinal absorption, contributes to pulsatile stimulation of dopamine receptors and the emergence of motor fluctuations and dyskinesias [5]. Similar mechanisms may also contribute to non-motor fluctuations and selected dopaminergic-responsive NMS, suggesting that unstable dopaminergic stimulation may influence both motor and non-motor manifestations of advanced PD [4,6]. This pathophysiological rationale supports therapeutic strategies aimed at providing more continuous dopaminergic stimulation and reducing fluctuations in clinical response.

During the advanced phase of PD, device-aided therapies (DATs) are frequently used to manage motor fluctuations and dyskinesias that are not satisfactorily controlled by oral therapies. Among DATs, continuous infusion treatments are commonly used in clinical practice. These include levodopa–carbidopa intestinal gel (LCIG), continuous subcutaneous apomorphine infusion (CSAI), and, more recently, subcutaneous levodopa formulations, including foslevodopa/foscarbidopa and ND0612 [7,8], as well as levodopa–entacapone–carbidopa intestinal gel (LECIG). By providing more continuous dopaminergic delivery, these therapies enable more stable dopaminergic stimulation and have demonstrated efficacy in reducing motor complications [9,10,11,12,13].

However, the impact of infusion therapies on NMS remains incompletely characterized. Most studies have primarily focused on motor outcomes, with NMS often reported as secondary or exploratory endpoints. As a result, available evidence on non-motor outcomes is frequently limited to selected domains, inconsistently assessed, or based on short-term observations. Furthermore, the use of heterogeneous assessment tools and the absence of a systematic evaluation encompassing the full spectrum of NMS have limited a comprehensive understanding of the overall non-motor effects of these therapies.

A previous systematic review showed that LCIG could be beneficial for cardiovascular, gastrointestinal and urinary symptoms of autonomic dysfunction in advanced PD [14]. Nevertheless, less is known about the effect of infusion therapies on other NMS and about the available evidence across different infusion modalities.

Given the increasing clinical relevance of infusion therapies and the central role of NMS in PD management, a comprehensive synthesis of the available evidence is warranted. Therefore, the aim of this systematic review was to evaluate the effects of continuous infusion therapies on NMS in patients with advanced PD, focusing on clinically meaningful outcomes across the full spectrum of non-motor domains.

## 2. Materials and Methods

### 2.1. Search Strategy and Eligibility Criteria

We conducted a systematic review in accordance with the Preferred Reporting Items for Systematic Reviews and Meta-analyses (PRISMA) guidelines on the available evidence of efficacy of infusion therapies on NMS in advanced PD patients (Table A1). This systematic review was prospectively registered in the International Prospective Register of Systematic Reviews (PROSPERO; registration number CRD420261379907). Because this was a systematic review of publications, ethical standards committee approval and patient informed consent were not applicable.

We searched the PubMed, Embase, and Cochrane databases for relevant articles using the following keywords: [“Parkinson disease” OR “Parkinson’s disease” OR “Parkinson”] AND [“levodopa infusion” OR “intrajejunal levodopa infusion” OR “levodopa carbidopa intestinal gel” OR “levodopa carbidopa intrajejunal infusion” OR “LCIG” OR “LECIG” OR “levodopa entacapone carbidopa intestinal gel” OR “intestinal gel” OR “duodenal infusion” OR “percutaneous endoscopic gastrostomy” OR “apomorphine infusion” OR “continuous subcutaneous apomorphine infusion” OR “CSAI” OR “subcutaneous levodopa” OR “continuous subcutaneous levodopa infusion” OR “CSLI” OR “foslevodopa” OR “foscarbidopa” OR “foslevodopa/foscarbidopa”] AND [“non-motor symptom*” OR “nonmotor symptom*” OR “depression” OR “depressive symptom*” OR “depressive disorder*” OR “mood disorder*” OR “anxiety” OR “anxiety disorder*” OR “apathy” OR “psychosis” OR “psychotic symptom*” OR “hallucination*” OR “delusion*” OR “sleep disorder*” OR “sleep disturbance*” OR “sleep dysfunction” OR “REM sleep behavior disorder” OR “RBD” OR “excessive daytime sleepiness” OR “daytime sleepiness” OR “hypersomnolence” OR “insomnia” OR “fragmented sleep” OR “fatigue” OR “mental fatigue” OR “physical fatigue” OR “pain” OR “chronic pain” OR “musculoskeletal pain” OR “central pain” OR “cognitive impairment” OR “cognitive dysfunction” OR “cognitive decline” OR “cognition” OR “mild cognitive impairment” OR “dementia” OR “impulse control disorder*” OR “impulse-control disorder*” OR “behavioral addiction*” OR “compulsive behavior*” OR “pathological gambling” OR “hypersexuality” OR “compulsive eating” OR “punding”].

Original articles published in peer-reviewed journals between January 2005 and 1 March 2026 were screened. Only articles published in the English language and involving human subjects were considered. Eligible studies included randomized controlled trials (RCTs), observational cohort studies with or without a control group, and case series. Case reports, letters to the editor, reviews, meta-analyses, abstracts, editorials, and book chapters were excluded.

Studies were included if (1) evaluated participants were diagnosed with PD and treated with an infusion therapy and (2) reported efficacy outcomes regarding NMS were assessed through validated rating scales or structured quantitative measures.

Studies were excluded if they (1) included individuals with alternative diagnoses to PD and (2) did not provide sufficient information to determine the direction of change (improvement, stability, or worsening) in NMS in comparison to the evaluation preceding the switch to infusion therapies. No restrictions were applied regarding patient’s clinical and demographic features.

Titles and abstracts were screened for thematic relevance. After the exclusion of duplicated and non-relevant articles, full-text articles were independently reviewed for eligibility criteria by two authors (LDC, CL). In case of disagreement, at least two senior authors (DR, GI) were consulted to achieve consensus. The reference list of each included article was searched to identify additional studies not captured by the original search strategy.

In cases of multiple publications from the same cohort, only the most recent study or the one providing the most comprehensive non-motor assessment was included.

### 2.2. Methodological Quality

Four authors (GI, DR, LDC, CL) independently performed the quality appraisal of the selected studies. The risk of bias in individual studies was evaluated using the National Heart, Lung, and Blood Institute tools (NHLBI Quality Appraisal Tools) following the Cochrane Handbook recommendations [15]. Studies were rated as poor, fair, or good using the recommended questionnaire-based analysis regarding study design, research question, sample size and selection, eligibility criteria, literature search, clarity of exposition, methods, description of results, potential confounding variables, and limitations [15]. Because this review was based on published aggregate data and no quantitative meta-analysis was performed, statistical adjustment for potential type I errors reported in individual studies was not feasible. To address this limitation, findings were interpreted in the context of methodological quality, risk-of-bias assessment, and consistency of results across independent studies.

Due to the qualitative design of the review and the marked heterogeneity in study designs, follow-up durations, and outcome measures, a formal quantitative assessment of publication bias or risk of bias across studies was not performed. Nevertheless, the predominance of observational open-label studies was considered during interpretation of the findings, given the potential risk of selective outcome reporting and publication bias.

### 2.3. Data Extraction

At the last visit prior to initiation of infusion therapy (baseline evaluation), we collected data on patients’ demographics, disease stage assessed using the Hoehn and Yahr scale in the ON state, and clinical rating scale scores used for NMS assessment. Patients’ clinical features, including number of hours of infusion per day and rating scale scores, were further collected at the last time point evaluated by the studies. Outcomes regarding NMS were categorized into predefined clinical domains, including global non-motor burden, sleep disorders, mood and neuropsychiatric symptoms, cognition, autonomic symptoms and fatigue.

The effect of infusion therapies on NMS was assessed through widely used and validated clinical scales, including the Non-Motor Symptoms Scale (NMSS), Movement Disorders Society Unified Parkinson’s Disease Rating Scale (MDS-UPDRS) part I, Scales for Outcomes in Parkinson’s Disease-Autonomic (SCOPA-AUT), and other domain-specific instruments (e.g., PDSS, ESS, BDI, HADS, MoCA, MMSE, QUIP-RS).

### 2.4. Data Analysis

Due to the methodological heterogeneity in the assessment and reporting of non-motor outcome measures in the included studies, we did not perform a meta-analysis. Given the heterogeneity of both study design and results presentation across reports, we performed a qualitative evaluation of efficacy of infusion therapies on NMS, analyzing significant improvement, stability, or worsening of NMS at follow-up in comparison to baseline. Results were reported as mean ± standard deviation (SD) or the median and range, as appropriate. Qualitative data were reported using frequency (percentage). When descriptive baseline and follow-up values were summarized across studies, values were reported as unweighted averages of study-level reported means and standard deviations and should therefore be interpreted descriptively rather than as pooled quantitative estimates.

Because some studies reported multiple treatment arms, such as different infusion therapies, or provided outcome data at multiple follow-up time points, each independent baseline-to-follow-up evaluation was considered as a separate comparison. Therefore, the total number of comparisons may exceed the number of included studies. This approach was adopted to capture all available outcome data while preserving the within-study structure of analyses.

When multiple outcome measures were used within the same non-motor domain in a single study, a single domain-level judgment (improvement, stability, or worsening) was assigned. Priority was given to the most specific and validated scale for that domain (e.g., MMSE or MoCA for cognition), while additional scales were considered to confirm consistency of direction. In case of discordant results, the domain-specific instrument was considered the primary reference. This domain-level synthesis approach was intended to provide a clinically oriented descriptive overview of non-motor outcomes across heterogeneous studies and assessment tools, rather than a quantitative estimate of treatment efficacy.

## 3. Results

### 3.1. Study Selection and Characteristics

**Figure 1:**
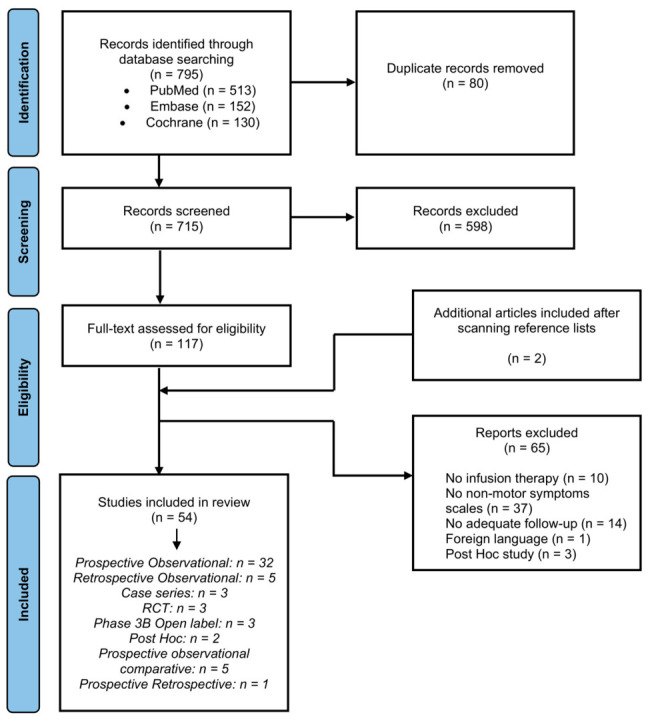
The PRISMA flow diagram of the systematic review.

Of the 795 records identified through database searching, 54 studies met the inclusion criteria and were included in the qualitative synthesis (Figure 1).

Among the included studies, 38 evaluated LCIG, 14 evaluated CSAI, six evaluated subcutaneous levodopa formulations (including one RCT regarding ND0612 and five studies on foslevodopa/foscarbidopa), and two evaluated LECIG. One study evaluated all four infusion therapies [16], while three studies included both LCIG and CSAI groups [12,17,18].

Regarding study quality appraisal, 11 studies were rated good, 34 were rated fair, and nine were rated poor. Most studies were prospective observational studies (*n* = 32), followed by prospective comparative studies (*n* = 5), retrospective observational studies (*n* = 5), case series (*n* = 3), randomized controlled trials (*n* = 3), phase 3 open-label studies (*n* = 3), and post hoc analyses (*n* = 2).

A total of 4157 patients were included at baseline, with follow-up data available for 2919 patients (2079 treated with LCIG, 403 treated with subcutaneous levodopa formulations, 381 treated with CSAI, 56 treated with LECIG). Baseline demographic and clinical characteristics were extracted when available. Sex distribution was reported for 3383 of 4157 patients, of whom 1983 were male (58.6%) and 1400 were female (41.4%). At baseline, mean age was 65.9 ± 8.4 years, and mean disease duration was 12.6 ± 5.0 years. Hoehn and Yahr stage in the ON state was available in 32 of 54 studies, with a mean score of 2.7 ± 0.7.

The average infusion therapy duration was 15.6 ± 5.1 months, with a maximum follow-up of 78.8 months for LCIG, 30 months for CSAI, 12 months for subcutaneous levodopa, and 7.3 months for LECIG. Infusion was typically administered 14–16 h per day. Night-time infusion was uncommon, reported only in 11 studies including LCIG, subcutaneous levodopa formulations and LECIG subgroups and in one CSAI study.

Baseline LEDD was reported in 36 studies (mean 1285.7 ± 329.2 mg), and follow-up LEDD was reported in 33 studies (mean 1532.1 ± 449.4 mg). Detailed study characteristics, including study-level sex distribution and Hoehn and Yahr stage in the ON state when available, are summarized in Table A2.

### 3.2. Overall Efficacy of Infusion Therapies on NMS

#### 3.2.1. Global Non-Motor Burden

Global non-motor burden was primarily assessed using the NMSS total score, reported in 27 studies for 33 comparisons (27 LCIG, five CSAI, one LECIG). Mean NMSS total score decreased from 84.4 ± 35.2 at baseline to 54.9 ± 17.6 at follow-up. Among studies reporting baseline-to-follow-up comparisons, significant improvement was observed in 28 out of 33 comparisons (85%), while no significant change was reported in five comparisons (15%), all involving LCIG-treated patients.

One additional study reported global non-motor outcomes using a non-validated composite score, showing significant improvement in patients treated with foslevodopa/foscarbidopa, CSAI, and LECIG but not with LCIG (improvement in three out of four comparisons between baseline and follow-up) [16].

MDS-UPDRS part I was assessed in six studies (four LCIG, one CSAI, one subcutaneous foslevodopa), showing a reduction from 14.8 ± 5.7 to 13.7 ± 5.0, with significant improvement in five out of eight comparisons (66.7%).

UPDRS part I was reported in nine studies (five LCIG, four CSAI) and showed less consistent results, with significant improvement in only two out of nine comparisons (22%).

Considering a domain-level synthesis prioritizing the most specific available measure for each study or comparison, global non-motor burden improved in 33 out of 45 baseline-to-follow-up comparisons (73.3%), remained stable in 11 (24.4%), and worsened in one (2.2%) (Table 1).

#### 3.2.2. Sleep-Related Symptoms and Fatigue

Sleep-related outcomes were among the most frequently assessed non-motor domains. PDSS-2 was reported in 16 studies (10 LCIG, four subcutaneous levodopa formulations, one CSAI, one LECIG), while PDSS was reported in five studies (four LCIG, one CSAI). Two additional studies reported outcomes using the Pittsburgh Sleep Quality Index.

PDSS-2 scores improved from 25.7 ± 4.7 to 20.1 ± 5.2, with significant improvement in 14 out of 16 comparisons (88%). PDSS scores improved in all five available comparisons. Pittsburgh Sleep Quality Index improved in two out of three comparisons (66.7%).

Consistently, NMSS Domain 2, assessing sleep and fatigue, showed the most robust improvement among NMSS domains, with significant improvement in 17 out of 20 comparisons (85%).

Excessive daytime sleepiness was assessed using the Epworth Sleepiness Scale in 10 studies (nine LCIG, one CSAI). ESS scores decreased from 8.8 ± 1.8 to 7.0 ± 2.1, with significant improvement in three out of 11 comparisons (27%).

Using the same domain-level approach, sleep and fatigue outcomes improved in 26 out of 31 comparisons (83.9%) and remained stable in five (16.1%), with no worsening reported.

Fatigue was evaluated using dedicated scales in a limited number of studies. The Fatigue Severity Scale and PFS-16 were each used in one study, showing no significant change and improvement, respectively.

#### 3.2.3. Mood and Neuropsychiatric Symptoms

Mood and neuropsychiatric symptoms were assessed using several scales. Depressive symptoms were evaluated using the Beck Depression Inventory in six studies, with improvement in one out of six comparisons (17%), worsening in one comparison, and no significant change in the remaining studies (67%).

Anxiety and mood-related symptoms were assessed using the State-Trait Anxiety Inventory (STAI), Beck Anxiety Inventory, and Hamilton scales in two, one, and five studies, respectively. Improvement was reported in zero out of two STAI comparisons, one out of one comparison using Beck Anxiety Inventory, and one out of six comparisons using Hamilton scale (17%).

NMSS Domain 3, assessing mood/cognition, improved in 12 out of 20 comparisons (60%).

Hallucinations and perceptual symptoms were evaluated through NMSS Domain 4, which improved in nine out of 19 comparisons (47%). Impulse control disorders were assessed using QUIP-RS in five studies and QUIP in one study. Improvement was reported in two out of five comparisons using QUIP-RS (40%) and in the single comparison using QUIP.

Overall, mood and neuropsychiatric outcomes improved in 14 out of 28 comparisons (50.0%) and remained stable in 14 (50.0%), with no domain-level worsening classification reported.

#### 3.2.4. Cognitive Function

Cognitive function was primarily assessed using MMSE in 19 studies, showing a slight overall decline from 27.2 ± 2.0 to 26.7 ± 2.9. Pre–post comparisons were available in 16 cohorts, with no significant changes reported in 11 and worsening reported in five.

Similar findings were observed with other cognitive scales. MoCA was used in four studies, with worsening reported in one study and no significant change reported in the remaining studies. FAB was used in six studies, with worsening in two and no significant change in the others. MDRS was used in five studies, with worsening in one and no significant change in the remaining studies. Collectively, the cognitive instruments used across studies primarily assessed global cognitive performance and executive functions rather than specific cognitive domains.

NMSS Domain 5, assessing attention/memory, improved in seven out of 18 comparisons (39%), and no significant changes were reported in the remaining.

Overall, cognitive function improved in seven out of 37 comparisons (18.9%), remained stable in 23 (62.2%), and worsened in seven (18.9%).

#### 3.2.5. Autonomic Symptoms

Autonomic symptoms were mainly captured through NMSS domains, while dedicated autonomic scales were rarely used. Only one study reported SCOPA-AUT outcomes, showing no significant change from baseline to follow-up.

Among NMSS autonomic-related domains, gastrointestinal symptoms (NMSS Domain 6) showed the most consistent improvement, observed in 14 out of 20 comparisons (70%). Urinary symptoms, assessed through NMSS Domain 7, showed improvement in nine out of 18 comparisons (50%). Cardiovascular symptoms, assessed through NMSS Domain 1, improved in five out of 18 comparisons (28%). Sexual function, assessed through NMSS Domain 8, showed the least consistent response, with improvement in only two out of 19 comparisons (11%).

#### 3.2.6. Other Non-Motor Domains

NMSS Domain 9, including miscellaneous NMS (including pain, excessive sweating, changes in weight and ability to smell or taste), improved in 11 out of 19 comparisons (57.9%). No study reported outcomes using pain-specific scales.

**Figure 2:**
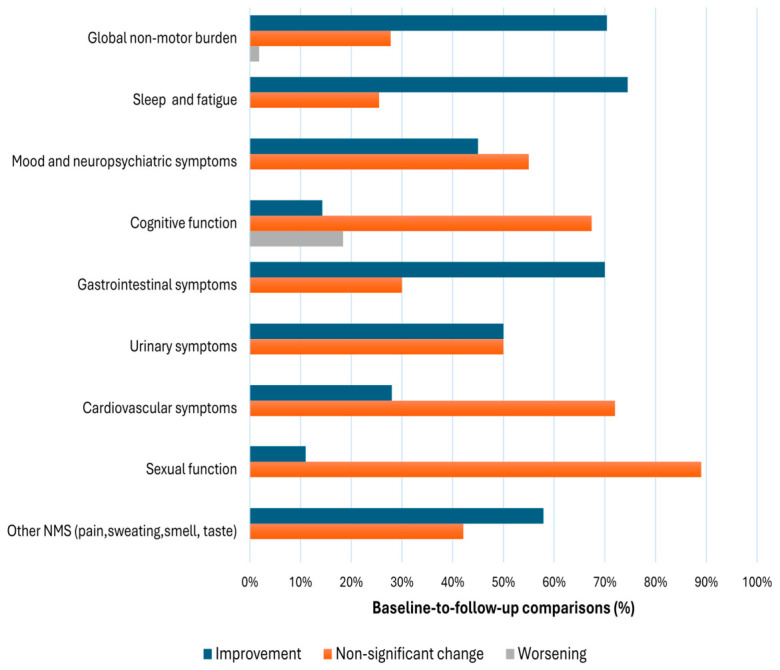
Proportion of baseline-to-follow-up comparisons showing improvement, stability, or worsening across non-motor domains in advanced Parkinson’s disease. Each bar represents the percentage of comparisons within a given domain classified according to direction of change. Comparisons refer to distinct baseline-to-follow-up evaluations, including different time points or treatment groups within the same study. The number of comparisons varies across domains depending on the availability of outcome data and the assessment tools used. NMS, non-motor symptoms.

**Figure A1:**
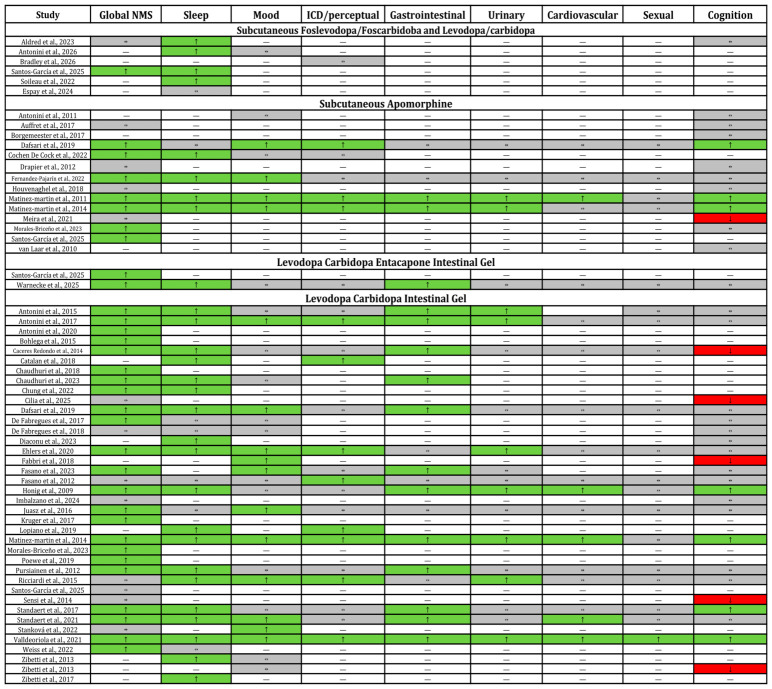
**Study-level heatmap of non-motor symptom outcomes across infusion therapies in advanced Parkinson’s disease.** Heatmap showing the direction of change in non-motor symptoms across studies and domains. Green (↑) improvement; Grey (↔) no significant change; Red (↓) worsening; White (—) not assessed. Only domain-specific outcomes are displayed; therefore, the number of comparisons differs from the total number reported for global non-motor burden.

Domain-specific outcome distributions are illustrated in Figure 2, while study-level patterns across non-motor domains are provided in Figure A1.

## 4. Discussion

NMS in Parkinson’s disease, particularly in advanced stages, are among the leading causes of disability, associated with a considerable burden for both patients and their caregivers; however, their management often remains suboptimal [3,18].

In this systematic review, we synthesized the available evidence on the efficacy of continuous infusion therapies on NMS in PD. Overall, infusion therapies were associated with an improvement in global non-motor burden in the majority of baseline-to-follow-up comparisons. However, treatment effects were heterogeneous across specific non-motor domains, with the most consistent benefits observed for sleep/fatigue and gastrointestinal symptoms, while cognitive, cardiovascular, sexual, and dedicated mood-related outcomes showed more limited or inconsistent effects.

A key finding of this review is that the available evidence is largely driven by studies investigating LCIG, which accounted for the majority of included cohorts and patients and the longest follow-up duration. Consequently, both global and domain-specific non-motor outcomes were more comprehensively evaluated for LCIG compared to other infusion therapies. Across LCIG studies, improvements were frequently observed in NMSS total score and in selected NMSS domains, particularly sleep/fatigue and gastrointestinal symptoms. These findings support the hypothesis that continuous dopaminergic delivery may reduce not only motor fluctuations but also non-motor fluctuations and dopaminergic-responsive NMS [3].

Evidence for CSAI was more limited and heterogeneous. Although several studies reported improvements in global non-motor burden and sleep-related outcomes, the number of studies assessing domain-specific outcomes was smaller, and results were less consistent. This variability likely reflects differences in study design, patient selection, and outcome measures and may also be related to differences in the pharmacological profile of apomorphine compared to levodopa-based formulations [19].

Data on subcutaneous levodopa formulations, including ND0612 and foslevodopa/foscarbidopa, remain limited. Available studies suggest possible improvements in global non-motor burden and sleep-related outcomes but are characterized by relatively small sample sizes, shorter follow-up durations, and limited domain-specific reporting. Importantly, given the continuous 24 h delivery of these therapies, future evidence may further elucidate their impact on nocturnal symptoms and other underexplored non-motor domains. As these therapies are increasingly adopted in clinical practice, more robust and longer-term studies, including standardized non-motor assessments, are needed.

Evidence on LECIG was scarce, with only two studies available. Preliminary findings suggest potential improvement in NMS, but the limited number of studies and short follow-up preclude firm conclusions. Further research is required to determine whether pharmacokinetic differences associated with entacapone co-administration translate into clinically meaningful non-motor benefits.

Among individual non-motor domains, sleep and fatigue showed the most consistent improvement. This was supported both by NMSS Domain 2 and by dedicated sleep scales, including PDSS and PDSS-2, which demonstrated improvement in the majority of comparisons. These findings are clinically relevant, as sleep disturbances in advanced PD are often related to nocturnal motor symptoms, non-motor fluctuations, and dopaminergic withdrawal [20]. In contrast, excessive daytime sleepiness, assessed using ESS, showed less consistent improvement, suggesting that this symptom may be influenced by additional factors, including disease severity, dopaminergic load, sleep fragmentation, and comorbidities [21].

Mood and neuropsychiatric outcomes showed greater heterogeneity across studies. While NMSS Domain 3 suggested improvement in a proportion of comparisons, dedicated mood scales, such as BDI, STAI, and Hamilton scales, showed limited and inconsistent changes. This discrepancy likely reflects differences in sensitivity and scope between global non-motor scales and symptom-specific psychiatric instruments. Broad scales such as the NMSS may capture overall changes in non-motor burden, including fluctuations and general well-being. In contrast, dedicated mood scales are designed to detect specific depressive or anxiety symptoms and may therefore be less sensitive to indirect or fluctuation-related improvements. From a clinical perspective, these findings suggest that mood symptoms may improve in some patients as a secondary effect of reduced motor and non-motor fluctuations. However, primary psychiatric manifestations in PD are likely multifactorial and may not be fully responsive to dopaminergic stabilization alone, thereby requiring targeted domain-specific therapeutic strategies [22].

Cognitive outcomes were largely stable, with worsening reported in a minority of comparisons. The available evidence does not suggest a consistent cognitive benefit from infusion therapies but neither does it indicate systematic worsening across studies. This is clinically important because cognitive impairment is a frequent feature in advanced PD populations and may even progress over time [23]. Nevertheless, the interpretation of cognitive outcomes is limited by heterogeneous tools, variable follow-up duration, and the frequent exclusion of patients with more severe cognitive impairment. Moreover, most studies relied on screening instruments, such as MMSE, MoCA, FAB, and MDRS, which predominantly assess global cognitive performance and executive functioning. While these measures are useful for detecting overall cognitive impairment, they provide limited information on individual cognitive domains. Therefore, the available evidence does not allow firm conclusions regarding the effects of infusion therapies on specific cognitive functions, including memory, attention, language, visuospatial abilities, and executive functions, as detailed neuropsychological assessments were rarely performed.

Autonomic symptoms showed mixed responses. Among these, gastrointestinal symptoms demonstrated the most consistent improvement, as reflected by NMSS Domain 6, which improved in the majority of comparisons. This may partly reflect improvement in gastrointestinal symptoms that are related to non-motor fluctuations and are therefore responsive to dopaminergic stimulation, such as abdominal discomfort, nausea, or bloating occurring during OFF periods. In this context, continuous dopaminergic delivery may reduce fluctuation-related gastrointestinal symptoms rather than directly improving structural or non-dopaminergic gastrointestinal dysfunction [24]. However, gastrointestinal outcomes were mostly captured through NMSS domains rather than dedicated gastrointestinal scales, limiting the ability to distinguish constipation, dysphagia, gastroparesis, abdominal discomfort, and other symptoms [25]. Given the relevance of gastrointestinal dysfunction for levodopa pharmacokinetics and clinical response, future studies should include more targeted assessments.

In contrast, other autonomic domains showed more limited or inconsistent responses. Cardiovascular symptoms, urinary dysfunction, and sexual function demonstrated modest or variable improvement, suggesting that these features may be less responsive to continuous dopaminergic stimulation and more related to non-dopaminergic or structural autonomic impairment [26].

The main strength of this review lies in the comprehensive synthesis of non-motor outcomes across both established and emerging infusion treatments. By organizing the findings according to clinically relevant non-motor domains, this review provides a structured overview of which symptoms appear most responsive and where evidence remains insufficient.

Importantly, the domains showing the most consistent improvement (sleep/fatigue and gastrointestinal symptoms) are among the most burdensome non-motor manifestations of advanced PD and are frequently associated with reduced quality of life, impaired daily functioning, and increased caregiver burden [1]. Therefore, the observed benefits may have clinical relevance beyond changes in rating scale scores. Improvement in sleep-related symptoms and fatigue may translate into better daytime functioning, greater social participation, and reduced caregiver demands. Likewise, improvement in gastrointestinal symptoms may have broader implications for disease management, as gastrointestinal dysfunction can adversely affect nutritional status, treatment adherence, and levodopa absorption, thereby contributing to fluctuations in clinical response [25]. Collectively, these findings suggest that the benefits of continuous infusion therapies may extend beyond motor control and contribute to aspects of health that are highly relevant to patients’ everyday lives.

Several limitations should be acknowledged. First, most included studies were observational, open-label, or non-randomized, with only a small number of RCTs. The predominance of observational and open-label studies may have increased the risk of selective outcome reporting and publication bias. Moreover, several included studies were based on relatively small samples and multiple exploratory outcome assessments, potentially increasing the risk of type I errors. Consequently, positive findings should be interpreted in the context of study quality and consistency of results across independent studies.

Second, NMS were often assessed as secondary or exploratory outcomes, leading to heterogeneous reporting and potential underestimation of treatment effects. Third, outcome measures varied widely across studies, limiting comparability. Broad composite instruments, such as the NMSS and MDS-UPDRS part I, are validated tools for capturing global non-motor burden, but they include heterogeneous domains and may differ in sensitivity to change across specific symptoms. Conversely, domain-specific instruments, such as sleep, mood, cognitive, autonomic, or impulse-control scales, may provide more reliable information for individual non-motor manifestations, but they were less consistently applied across studies. Therefore, differences in scale validity, domain coverage, and responsiveness may have influenced the direction and magnitude of reported treatment effects. Accordingly, the domain-level synthesis adopted in this review should be interpreted as a clinically oriented descriptive overview rather than as a quantitative estimate of treatment efficacy or as a direct comparison across instruments or treatment modalities.

Fourth, follow-up durations differed substantially between therapies, particularly for newer subcutaneous levodopa formulations and LECIG. Finally, the evidence was unbalanced across treatments, with a predominance of LCIG studies, limiting the ability to perform direct comparisons.

Future studies should adopt standardized and comprehensive assessment of NMS, combining global scales, such as NMSS or MDS-UPDRS part I, with domain-specific instruments for sleep, autonomic symptoms, mood, cognition, pain, fatigue, and impulse control disorders. Longer follow-up is particularly needed for newer infusion therapies. Comparative studies between infusion therapies and other device-aided treatments would also be valuable to guide clinicians in selecting the most appropriate option for advanced PD, taking into account the patient’s specific non-motor profile [27].

## 5. Conclusions

In conclusion, continuous infusion therapies appear to be associated with improvement in global non-motor burden in advanced PD, with the most consistent benefits observed for sleep/fatigue and gastrointestinal symptoms. Evidence is strongest for LCIG, whereas data for CSAI, LECIG, and subcutaneous levodopa formulations remain limited. Effects on cognition, cardiovascular and urinary symptoms, sexual function, and mood-specific outcomes are less consistent. Notably, although advanced therapies in PD are primarily tailored to address motor fluctuations and complications, these findings underscore the clinical relevance of systematically evaluating and managing non-motor symptoms.

## Figures and Tables

**Table 1:** Domain-level synthesis of non-motor outcomes across infusion therapies in advanced Parkinson’s disease.

Domain	Studies (n) *	Comparisons (n)	Therapies Evaluated, n Comparisons	Improvement, n (%)	Stability, n (%)	Worsening, n (%)	Main Assessment Tools	Overall Consistency **
Global non-motor burden	27	45	LCIG (31), CSAI (10), LECIG (2), CSFLI (2)	33 (73.3%)	11 (24.4%)	1 (2.2%) ***	NMSS, MDS-UPDRS I, UPDRS I	High
Sleep and fatigue	19	31	LCIG (21), CSAI (5), CSFLI (3), ND0612 (1), LECIG (1)	26 (83.9%)	5 (16.1%)	0	PDSS-2, PDSS, ESS, PSQI, NMSS domain 2	High
Mood and neuropsychiatric symptoms	17	28	LCIG (20), CSAI (6), CSFLI (1), LECIG (1)	14 (50.0%)	14 (50.0%)	0	ALS, BDI, STAI, Hamilton, QUIP-RS, QUIP, NMSS domain 3–4	Moderate–low
Cognitive function	19	37	LCIG (22), CSAI (13), CSFLI (1), LECIG (1)	7 (18.9%)	23 (62.2%)	7 (18.9%)	MMSE, MoCA, FAB, MDRS, NMSS domains 3–5	Low
Gastrointestinal symptoms	18	20	LCIG (15), CSAI (4), LECIG (1)	14 (70%)	6 (30%)	0	NMSS domain 6	High
Urinary symptoms	18	18	LCIG (14), CSAI (4)	9 (50%)	9 (50%)	0	NMSS domain 7	Moderate
Cardiovascular symptoms	18	18	LCIG (13), CSAI (4), LECIG (1)	5 (28%)	13 (72%)	0	NMSS domain 1	Low
Sexual function	17	19	LCIG (14), CSAI (4), LECIG (1)	2 (11%)	17 (89%)	0	NMSS domain 8	Low
Other NMS (pain, sweating, smell, taste)	18	19	LCIG (14), CSAI (5)	11 (57.9%)	8 (42.1%)	0	NMSS domain 9	Moderate

When multiple outcome measures were used within the same non-motor domain in a single study, a single domain-level judgment (improvement, stability, or worsening) was assigned. Priority was given to the most specific and validated scale for that domain (e.g., MMSE or MoCA for cognition rather than NMSS domain scores), while additional scales were considered to confirm consistency of direction. In case of discordant results, the domain-specific instrument was considered the primary reference. * Study designs varied across domains and included prospective observational studies, retrospective observational studies, comparative observational studies, randomized controlled trials, phase 3 open-label studies, post hoc analyses, mixed prospective–retrospective studies, and case series. ** Overall consistency was descriptively categorized according to the proportion of baseline-to-follow-up comparisons showing improvement within each domain. These thresholds were defined a priori for descriptive purposes only and should not be interpreted as formal measures of evidence certainty: High: ≥70% of comparisons showing improvement; Moderate: 40–69%; Low: <40%. *** Worsening in global non-motor burden was observed only in one long-term follow-up comparison using MDS-UPDRS part I. ALS, Lille Apathy scale; BDI, Beck Depression Inventory; CSFLI, Continuous subcutaneous infusion of foslevodopa/foscarbidopa; ESS, Epworth Sleepiness Scale; FAB, Frontal Assessment Battery; MDRS, Mattis Dementia Rating Scale; MDS-UPDRS I, Movement Disorder Society–Unified Parkinson’s Disease Rating Scale Part I; MMSE, Mini-Mental State Examination; MoCA, Montreal Cognitive Assessment; ND0612, continuous subcutaneous levodopa/carbidopa infusion; NMSS, Non-Motor Symptoms Scale; PDSS, Parkinson’s Disease Sleep Scale; PDSS-2, Parkinson’s Disease Sleep Scale–2; PSQI, Pittsburgh Sleep Quality Index; QUIP-RS, Questionnaire for Impulsive-Compulsive Disorders in Parkinson’s Disease–Rating Scale; STAI, State-Trait Anxiety Inventory; UPDRS I, Unified Parkinson’s Disease Rating Scale Part I.

## Data Availability

No new data were created or analyzed in this study. Data sharing is not applicable to this article.

## Appendix A

**Table A1:** Checklist PRISMA.

Section/Topic	#	Checklist Item	Reported on Page
TITLE			
Title	1	Identify the report as a systematic review, meta-analysis, or both.	1
ABSTRACT			
Structured summary	2	Provide a structured summary including, as applicable, background; objectives; data sources; study eligibility criteria, participants, and interventions; study appraisal and synthesis methods; results; limitations; conclusions and implications of key findings; and systematic review registration number.	1
INTRODUCTION			
Rationale	3	Describe the rationale for the review in the context of what is already known.	2
Objectives	4	Provide an explicit statement of questions being addressed with reference to participants, interventions, comparisons, outcomes, and study design (PICOS).	2
METHODS			
Protocol and registration	5	Indicate if a review protocol exists, if and where it can be accessed (e.g., Web address), and, if available, provide registration information including registration number.	4
Eligibility criteria	6	Specify study characteristics (e.g., PICOS, length of follow-up) and report characteristics (e.g., years considered, language, publication status) used as criteria for eligibility, giving rationale.	2–3
Information sources	7	Describe all information sources (e.g., databases with dates of coverage, contact with study authors to identify additional studies) in the search and date last searched.	2–3
Search	8	Present full electronic search strategy for at least one database, including any limits used, such that it could be repeated.	2–3
Study selection	9	State the process for selecting studies (i.e., screening, eligibility, included in systematic review, and, if applicable, included in the meta-analysis).	3–4
Data collection process	10	Describe method of data extraction from reports (e.g., piloted forms, independently, in duplicate) and any processes for obtaining and confirming data from investigators.	4
Data items	11	List and define all variables for which data were sought (e.g., PICOS, funding sources) and any assumptions and simplifications made.	4
Risk of bias in individual studies	12	Describe methods used for assessing risk of bias of individual studies (including specification of whether this was done at the study or outcome level) and how this information is to be used in any data synthesis.	3–4
Summary measures	13	State the principal summary measures (e.g., risk ratio, difference in means).	4
Synthesis of results	14	Describe the methods of handling data and combining results of studies, if done, including measures of consistency (e.g., I^2^) for each meta-analysis.	4
Risk of bias across studies	15	Specify any assessment of risk of bias that may affect the cumulative evidence (e.g., publication bias, selective reporting within studies).	4
Additional analyses	16	Describe methods of additional analyses (e.g., sensitivity or subgroup analyses, meta-regression), if done, indicating which were pre-specified.	NA
RESULTS			
Study selection	17	Give numbers of studies screened, assessed for eligibility, and included in the review, with reasons for exclusions at each stage, ideally with a flow diagram.	5
Study characteristics	18	For each study, present characteristics for which data were extracted (e.g., study size, PICOS, follow-up period) and provide the citations.	18–22
Risk of bias within studies	19	Present data on risk of bias of each study and, if available, any outcome level assessment (see item 12).	18–22
Results of individual studies	20	For all outcomes considered (benefits or harms), present for each study (a) simple summary data for each intervention group and (b) effect estimates and confidence intervals, ideally with a forest plot.	18–22
Synthesis of results	21	Present results of each meta-analysis done, including confidence intervals and measures of consistency.	6–12
Risk of bias across studies	22	Present results of any assessment of risk of bias across studies (see Item 15).	14
Additional analysis	23	Give results of additional analyses, if done (e.g., sensitivity or subgroup analyses, meta-regression [see Item 16]).	NA
DISCUSSION			
Summary of evidence	24	Summarize the main findings, including the strength of evidence for each main outcome; consider their relevance to key groups (e.g., healthcare providers, users, and policy makers).	12–14
Limitations	25	Discuss limitations at study and outcome level (e.g., risk of bias) and at review-level (e.g., incomplete retrieval of identified research, reporting bias).	14
Conclusions	26	Provide a general interpretation of the results in the context of other evidence and implications for future research.	15
FUNDING			
Funding	27	Describe sources of funding for the systematic review and other support (e.g., supply of data) and role of funders for the systematic review.	15

**Table A2:** Characteristics of studies evaluating infusion therapies on non-motor symptoms in advanced Parkinson’s disease.

Study	Study Design	Sample Size (n, Men/Women)	H and Y	Therapy	Follow-Up (Months)	Mean Age at Infusion Initiation (years)	Disease Duration at Infusion Initiation (Years)	NMS Assessment Tools	Study Quality
Aldred et al., 2023 [7]	Phase 3 Open label	244 (146/98)	2.2 ± 0.7	CSFLI	12	63.9 ± 9.2	10.7 ± 5.2	MDS-UPDRS I, MMSE, PDSS-2	Good
Antonini et al., 2011 [28]	Prospective observational comparative	12 (7/5)	NA	CSAI	12/30	58 ± 12	NR	MMSE, HDS	Fair
Antonini et al., 2015 [29]	Prospective observational	172 (76/96)	2.8 ± 0.8	LCIG	12	66.5 ± 9.3	12.6 ± 6.6	NMSS	Fair
Antonini et al., 2017 [10]	Prospective observational	64 (39/25)	NA	LCIG	10.2 ± 4.3	70.4 ± 7.8	13.9 ± 5.4	NMSS	Fair
Antonini et al., 2020 [30]	Prospective observational	60 (37/23)	2.7 ± 0.6	LCIG	12/24	68.3 ± 8.1	11.7 ± 5.8	NMSS	Poor
Antonini et al., 2026 [31]	Post hoc of a phase III	13 (11/2)	NA	CSFLI	3	56.7 ± 8.4	NA	PDSS-2, STAI	Fair
Auffret et al., 2017 [32]	Case series	12 (7/5)	2.3 ± 0.6	CSAI	6	65.9 ± 7.2	13.8 ± 3.6	UPDRS I, MDRS, ALS	Fair
Bohlega et al., 2015 [33]	Prospective observational	18 (12/6)	NA	LCIG	6	52.6 ± 12.2	11.4 ± 4.2	NMSS	Fair
Borgemeester et al., 2017 [34]	Retrospective observational	45 (26/19)	3.2 ± 1.1	CSAI	2	70.9 ± 8.1	10.8 ± 4.8	MMSE, FAB, QUIP-RS	Poor
Bradley et al., 2026 [35]	Case series	5 (5/0)	NA	CSFLI	4.8 ± 1.6	59 ± 8.1	16 ± 1.6	QUIP-RS	Fair
Cáceres-Redondo et al., 2014 [36]	Retrospective observational	29 (16 available, 7/9)	NA	LCIG	32.2 ± 12.4	64.5 ± 9	14.1 ± 3.9	NMSS, MMSE	Good
Catalan et al., 2018 [37]	Prospective observational	62 (36/26)	3	LCIG	6	71.2 ± 7	12.8 ± 5.8	MMSE, PDSS, ESS, QUIP-RS	Poor
Chaudhuri et al., 2019 [38]	Post hoc analysis of prospective observational	233 (NA)	NA	LCIG	24	66.2 ± 8.5	12.5 ± 5.9	NMSS	Fair
Chaudhuri et al., 2023 [39]	Prospective observational	195 (120/75)	2.9 ± 0.7	LCIG	36	70.2 ± 8.2	11.2 ± 4.8	NMSS, MMSE, PDSS-2	Fair
Chung et al., 2022 [40]	Phase 3 Open label	43 (29/14)	NA	LCIG	6.5	66.9 ± 7.3	11.7 ± 4.9	NMSS, PDSS-2	Fair
Cilia et al., 2025 [41]	Retrospective observational	512 (NA)	2.8 ± 0.8	LCIG	12.6 ± 1.4/58.5 ± 6.1	67 ± 8	12.9 ± 5	MDS-UPDRS I, MoCA	Good
Dafsari et al., 2019 [12]	Prospective observational	33 (16/17)/39 (17/22)	3.3 ± 0.7	LCIG/CSAI	6/6	65.4 ± 8.8/61.6 ± 9.7	14.6 ± 5.3/13.5 ± 5.6	NMSS	Good
Cochen De Cock et al., 2022 [42]	RCT double-blind crossover	46 (27/19)	NA	CSAI	0.6	63.6 ± 9.2	9.8 ± 4.5	MDS-UPDRS I, PDSS, ESS, STAI, BDI, HDS, QUIP-RS	Fair
De Fabregues et al., 2017 [43]	Prospective observational	37 (22/15)	NA	LCIG	6	68.2 ± 6.8	13.5 ± 5.6	UPDRS I, MMSE, ESS, BDI, HDS, HAS, FS	Fair
De Fabregues et al., 2018 [44]	Prospective observational	7 (1/6)	NA	LCIG	6	69.6	NA	UPDRS I, MMSE, ESS, BDI, HDS, HAS, FS, PSQI	Poor
Diaconu et al., 2023 [45]	Prospective observational	10 (7/3)	3.6 ± 0.5	LCIG	6/12	69.8 ± 8.4	7 ± 2.7	MMSE, PDSS-2, ESS, PSQI	Fair
Drapier et al., 2012 [46]	Prospective observational	23 (16/7))	2.3 ± 0.9	CSAI	12	NA	13.9 ± 8.2	UPDRS I, MDRS	Poor
Ehlers et al., 2020 [47]	Prospective observational	12 (7/5)	3	LCIG	6	68	16 ± NA	NMSS, HADS	Fair
Espay et al., 2024 [8]	RCT	128 (85/43)	< 3	ND0612	3	63.5 ± 9.5	9.30 ± 3.8	PDSS-2	Good
Fabbri et al., 2019 [48]	Prospective observational	44 (29/15)	3 ± 0.9	LCIG	51.6 ± 28.5	67.4 ± 5.8	14 ± 5.8	MMSE, BDI, HDS	Fair
Fasano et al., 2023 [49]	Retrospective cross-sectional	387 (252/135)	NA	LCIG	16.6 ± 3.9/30.3 ± 3.3/41.8 ± 3.7/53.7 ± 3.3/78.8 ± 18.6	67.9 ± 7.4/66.2 ± 8.4/65.8 ± 7.5/64.5 ± 7.6/65 ± 8.2	12.1 ± 5.4/13 ± 5.4/13.7 ± 5.4/12.2 ± 5.7/13.8 ± 5.1	UPDRS I, NMSS, MMSE, PDSS-2	Fair
Fasano et al., 2012 [50]	Retrospective observational	14 (10/4)	NA	LCIG	24.9	67.1 ± 11.5	12.9 ± 4.8	UPDRS I, NMSS MMSE, FAB PDSS QUIP	Fair
Fernandez-Pajarín et al., 2022 [51]	Prospective observational	22 (11/11)	NA	CSAI	6	59.4 ± 6.1	8.7 ± 3.5	NMSS, PDSS-2, QUIP-RS, MDRS	Good
Honig et al., 2009 [52]	Prospective observational	22 (16/6)	3.1 ± 0.7	LCIG	6	58.6 ± 9.1	15.3 ± 5.9	NMSS, PDSS	Fair
Houvenaghel et al., 2018 [53]	Prospective observational	22 (8/14)	1.4 ± 0.9	CSAI	6	57.5 ± 9.7	11.1 ± 4.4	UPDRS I, MDRS	Good
Imbalzano et al., 2024 [54]	Prospective observational	12 (7/5)	2.4 ± 0.9	LCIG	6	66 ± 9	11.1 ± 2.1	MDS-UPDRS I, MoCA	Fair
Juhász et al., 2017 [55]	Prospective observational	34 (19/15)	2.9 ± 0.8	LCIG	12	67 ± 6	12 ± 5	NMSS, MDS-UPDRS I, MMSE, MoCA, PDSS-2, ESS	Poor
Krüger et al., 2017 [56]	Prospective observational	58 (30/28)	NA	CSAI	12	70.4 ± 7.8	13.9 ± 5.4	NMSS	Good
Lopiano et al., 2019 [57]	Prospective, retrospective	145 (73/72)	NA	LCIG	12	70.4 ± 7.7	14.61 ± 6.58	PDSS-2. QUIP-RS	Good
Martinez-martin et al., 2011 [58]	Prospective observational comparative	17 (NA)	3.9	LCIG	12	59.5 ± 11.7	12.5 ± 4	NMSS	Fair
Martinez-martin et al., 2015 [18]	Prospective observational	44 (24/20)/43 (23/20)	3.5	LCIG/CSAI	6/6	62.7 ± 9.1/62.3 ± 10.6	16.1 ± 6.7/14 ± 4.5	NMSS	Fair
Meira et al., 2021 [59]	Prospective observational	110 (55/55)	NA	LCIG	24	62.9 ± 9.6	12 ± 6.1	UPDRS I, MMSE, FAB	Fair
Morales-Briceño et al., 2023 [17]	Prospective observational	13 (NA)/19 (NA)	1.8	CSAI/LCIG	12/12	60.5 (SD NR)/67 (SD NR)	10/13	NMSS, MDS-UPDRS I, MoCA	Fair
Poewe et al., 2019 [60]	Prospective observational	80 (45/35)/47 (28/19)/81 (52/29)	NA	LCIG	24/24/24	67.1/66.6/67.1	13.8/12.9/12.9	NMSS	Fair
Pursiainen et al., 2012 [61]	Case series	9 (9/0)	NA	LCIG	2	68.5 ± 6.2	12.5 ± 2.2	NMSS	Fair
Ricciardi et al., 2016 [62]	Prospective observational	8 (7/1)	NA	LCIG	36 ± 22	65 ± 6.9	14 ± 4.7	NMSS, MMSE, PDSS	Fair
Santos-García et al., 2025 [16]	Prospective observational comparative	98 (60/38)/43 (25/18)/7 (6/1)/23 (13/10)	2	CSFLI/CSAI/LCIG/LECIG	5.7 ± 1.5/6 ± 1.5/7.6 ± 1.1/6.3 ± 1.5	68.5 ± 8.7/67.9 ± 8.9/62.4 ± 16.5/71.9 ± 8.2	14.5 ± 10.3/10.7 ± 3.9/9.9 ± 2.9/12.4 ± 3.3	NMS score	Fair
Sensi et al., 2014 [63]	Prospective observational	28 (10/18)	3.2 ± 0.7	LCIG	32.4 ± 9.4	67.6 ± 6.1	15.5 ± 4	NMSS, MMSE, FAB, PDSS-2	Fair
Soileau et al., 2022 [11]	Randomized, double-blind, double-dummy, active-controlled, multicenter, phase 3 trial	74 (50/28)	2.3 ± 0.7	CSFLI	3	66.3 ± 9.2	8.4 ± 4.2	PDSS-2	Fair
Standaert et al., 2017 [64]	Open-label phase 3b study	38 (22/16)	NA	LCIG	14.3 ± 6.3	64.3 ± 10.2	11.5 ± 5.3	UPDRS I, NMSS	Fair
Standaert et al., 2021 [65]	Prospective observational	195 (120/75)	3 ± 0.8	LCIG	16.5 ± 8.3	70.2 ± 8.2	11.20 ± 4.8	NMSS, MMSE, PDSS-2, ESS	Good
Stanková et al., 2022 [66]	Prospective observational comparative	9 (8/1)	3	LCIG	6	68.4 ± 5.9	10.30 ± 2	SCOPA-AUT	Poor
Valldeoriola et al., 2021 [67]	Prospective observational	59 (36/23)	1.7 ± 0.4	LCIG	6 ± 0.5	67.9 ± 7.5	12.70 ± 6	NMSS, BDI, PFS16	Good
van Laar et al., 2010 [68]	Prospective observational	10 (7/3)	3.1 ± 0.6	CSAI	1.5	67	13.00 ± NR	MMSE, FAB	Poor
Warnecke et al., 2025 [69]	Prospective observational	167 (99/68)	3.2 ± 0.8	LECIG	7.3 ± 3.4	68.2 ± 7.7	13.30 ± 6.3	NMSS, PDSS-2	Fair
Weiss et al., 2022 [70]	Prospective observational comparative	73 (41/32)	3.6 ± 0.8	LCIG	12 ± 1	72.9 ± 6.3	14.60 ± 7	NMSS, PDSS-2 ESS	Fair
Zibetti et al., 2013 [71]	Prospective observational	12 (8/4)	NA	LCIG	3.5 ± 2.4	71.7 ± 4.8	16.90 ± 4.4	PDSS-2, ESS	Fair
Zibetti et al., 2013 [72]	Prospective observational	25 (16/9)	NA	LCIG	36.2 ± 11.2	69.9 ± 5.8	12.10 ± 4.1	MMSE, FAB, BDI	Fair
Zibetti et al., 2017 [73]	Prospective observational	11 (8/3)	NA	LCIG	3.8 ± 1.2	69.7 ± 5.6	14.80 ± 4.5	PDSS-2, ESS	Fair

ALS, Lille Apathy Scale; BDI, Beck Depression Inventory; CSAI, continuous subcutaneous apomorphine infusion; CSFLI, continuous subcutaneous foslevodopa/foscarbidopa infusion; ESS, Epworth Sleepiness Scale; FAB, Frontal Assessment Battery; HADS, Hospital Anxiety and Depression Scale; HDS, Hamilton Depression Scale; HAS, Hamilton Anxiety Scale; LCIG, levodopa–carbidopa intestinal gel; LECIG, levodopa–entacapone–carbidopa intestinal gel; MDRS, Mattis Dementia Rating Scale; MDS-UPDRS I, Movement Disorder Society Unified Parkinson’s Disease Rating Scale Part I; MMSE, Mini-Mental State Examination; MoCA, Montreal Cognitive Assessment; NMSS, Non-Motor Symptoms Scale; PDSS, Parkinson’s Disease Sleep Scale; PDSS-2, Parkinson’s Disease Sleep Scale–2; PFS-16, Parkinson Fatigue Scale-16; PSQI, Pittsburgh Sleep Quality Index; QUIP-RS, Questionnaire for Impulsive-Compulsive Disorders in Parkinson’s Disease–Rating Scale; RCT, randomized controlled trial; SCOPA-AUT, Scales for Outcomes in Parkinson’s Disease–Autonomic; STAI, State-Trait Anxiety Inventory; UPDRS I, Unified Parkinson’s Disease Rating Scale Part I.
